# Heterozygous CX3CR1 Deficiency in Microglia Restores Neuronal β-Amyloid Clearance Pathways and Slows Progression of Alzheimer's Like-Disease in PS1-APP Mice

**DOI:** 10.3389/fimmu.2019.02780

**Published:** 2019-12-02

**Authors:** Suzanne E. Hickman, Elizabeth K. Allison, Uwanda Coleman, Nathan D. Kingery-Gallagher, Joseph El Khoury

**Affiliations:** ^1^Center for Immunology and Inflammatory Diseases, Massachusetts General Hospital and Harvard Medical School, Charlestown, MA, United States; ^2^Division of Infectious Diseases, Massachusetts General Hospital and Harvard Medical School, Charlestown, MA, United States

**Keywords:** microglia, Alzheimer's disease, CX3CR1, Aβ-degrading enzymes, fractalkine, chemokines

## Abstract

CX3CR1 is a chemokine receptor expressed on microglia that binds Fractalkine (CX3CL1) and regulates microglial recruitment to sites of neuroinflammation. Full deletion of CX3CR1 in mouse models of Alzheimer's disease have opposing effects on amyloid-β and tau pathologies raising concerns about the benefits of targeting CX3CR1 for treatment of this disease. Since most therapies achieve only partial blockade of their targets, we investigated the effects of partial CX3CR1 deficiency on the development and progression of amyloid-β deposition in the PS1-APP Alzheimer's mouse model. We generated PS1-APP mice heterozygous for CX3CR1 (PS1-APP-CX3CR1^+/−^) and analyzed these mice for Alzheimer's-like pathology. We found that partial CX3CR1 deficiency was associated with a significant reduction in Aβ levels and in senile-like plaque load in the brain as compared with age-matched PS1-APP mice. Reduced Aβ level in the brain was associated with improved cognitive function. Levels of the neuronal-expressed Aβ-degrading enzymes insulysin and matrix metalloproteinase 9, which are reduced in the brains of regular PS1-APP mice, were significantly higher in PS1-APP-CX3CR1^+/−^ mice. Our data indicate that lowering CX3CR1 levels or partially inhibiting its activity in the brain may be a therapeutic strategy to increase neuronal Aβ clearance, reduce Aβ levels and delay progression of Alzheimer's-Like disease. Our findings also suggest a novel pathway where microglial CX3CR1 can regulates gene expression in neurons.

## Introduction

Deposition of amyloid-β (Aβ) in the brain is one of the pathological hallmarks of Alzheimer's disease (AD). Aβ levels in the brain are regulated by several mechanisms: (1) the pathways that generate Aβ consisting of β-secretase and the γ-secretase complex (1); (2) the enzymes that clear Aβ by means of degradation, such as neprilysin, insulysin (IDE), and matrix metalloproteinase 9 (MMP9) (2, 3); (3) clearance of Aβ through phagocytosis by mononuclear phagocytes/microglia and astrocytes, and (4) transport across the blood brain barrier (4). Progression of Alzheimer's disease is associated with reduced Aβ-clearing pathways resulting in Aβ accumulation (4–6).

Chemokines are chemotactic cytokines that control recruitment of mononuclear phagocytes to sites of inflammation (7). CX3CR1 is a chemokine receptor expressed on microglia and a subset of monocytes (8, 9). CX3CR1 and its chemokine ligand fractalkine (CX3CL1) have been implicated in the recruitment of mononuclear phagocytes to sites of inflammation and injury (7, 10, 11) and play roles in the pathogenesis of several inflammatory conditions, including atherosclerosis (12, 13), neuropathic pain (14), and asthma (15). In Alzheimer's disease complete deletion of CX3CR1 in models of amyloid deposition reduced Aβ deposits and enhanced microglial Aβ phagocytic ability (16, 17). These studies provided important insight on the role of CX3CR1 in AD and suggested that targeting CX3CR1 activity may be a therapeutic strategy to lower Aβ levels. It is unlikely that pharmacologic targeting of CX3CR1 will achieve the effects of complete deletion of this receptor as in CX3CR1^−/−^ mice. However, it is possible to achieve partial inhibition of CX3CR1 activity pharmacologically, similar to what is observed in mice heterozygous for this receptor (CX3CR1^+/−^).

To investigate the effect of partial CX3CR1 deficiency on progression of AD, we used bigenic mice expressing mutation of presenilin 1 and the amyloid precursor protein with the Swedish mutation (PS1-APP) (18, 19), and analyzed Alzheimer's-like pathology in PS1-APP mice heterozygous for CX3CR1 (PS1-APP-CX3CR1^+/−^). We found that partial CX3CR1 deficiency reduced plaque load and Aβ levels in the brains and mitigated the memory deficit in PS1-APP mice. This was associated with increased levels of the neuronal-expressed Aβ-degrading enzymes insulysin and MMP9, suggesting that partial microglial CX3CR1 deficiency restores the ability of neurons to clear Aβ. Since neuronal fractalkine is the only known ligand for CX3CR1, our results also suggest a novel pathway through which microglia can regulate neuronal gene expression possibly via CX3CR1-fractalkine interactions.

## Materials and Methods

### Mice

PS1-APP transgenic mice (B6C3-Tg (APPswe, PSEN1dE9)85Dbo/J stock number 004462) were purchased from The Jackson Laboratories and subsequently bred in the animal care facilities at Massachusetts General Hospital to a C57BL6 background. These mice co-express two genetic mutations that are associated with familial AD: a “humanized” Swedish amyloid precursor protein mutation (APP695SWE) and a mutant exon-9-deleted variant of human presenilin 1 (PSEN1/dE9). The APP and PSEN1 transgenes are integrated into a single locus and are independently under the control of separate mouse prion protein promoter elements, which direct expression of the transgenes predominantly to central nervous system neurons (18, 19).

CX3CR1-GFP mice were a generous gift from Dan Littman (New York University, New York). In this mouse strain, the CX3CR1 gene has been replaced by Green Fluorescent protein (GFP) reporter gene (20) and is maintained in the animal care facilities at Massachusetts General Hospital on a C57BL6 background. For our experiments, PS1-APP heterozygous mice were crossed with C57Bl/6 to generate PS1-APP heterozygous progeny and their WT littermates. In parallel breeding, CX3CR1^−/−^ mice were crossed with PS1-APP heterozygous mice and the resulting pups were genotyped at the time of weaning to select PS1-APP-CX3CR1^+/−^ genotypes. PS1-APP-CX3CR1^+/−^ F1 mice were also bred with CX3CR^−/−^ mice to obtain the desired genotypes PS1-APP-CX3CR1^+/−^, PS1-APP-CX3CR1^−/−^, WT-CX3CR1^+/−^, and WT-CX3CR1^−/−^. PS1-APP-CX3CR1^+/−^ mice are heterozygous for CX3CR1 and PS1-APP-CX3CR1^−/−^ mice have no functional CX3CR1 and both genotypes express GFP. In both genotypes, monocytes and microglia appear green under fluorescent microscopy. Because of the limited number of mice that can be generated, only male mice were used at ages ranging from 5 to 24 months and euthanized according to approved institutional procedures. All protocols were approved by the Massachusetts General Hospital Institutional Animal Care and Use Committee and met US National Institutes of Health guidelines for the humane care of animals.

### Immunofluorescence Staining for Total Aβ-Containing Plaques

Brains were harvested from transgenic PS1-APP, their WT littermates, PS1-APP-CX3CR1^−/−^ or PS1-APP-CX3CR1^+/−^ and fixed in 2% paraformaldehyde (PFA) in phosphate buffered saline (PBS) (Mediatech Inc., Manassas, VA) pH 7.5 overnight at 4°C. The fixed brains were then placed in 30% sucrose overnight at 4°C for cryoprotection. Brains were embedded in Tissue-Tek O.C.T compound (Sakura Finetek USA Inc., Torrance, CA) and cut into 10–12 μm frozen sections. To stain plaques of all sizes, sections were blocked with PBS/0.3%Triton-X 100/2% goat serum for 30 min, then incubated at 25°C overnight with rabbit anti-Aβ pan antibody (Life Technologies, Grand Island, NY) in 0.3% Triton X-100 and 2% goat serum. The slides were rinsed three times in PBS/0.3% TritonX-100 and then incubated with anti-rabbit IgG coupled to Alexa^555^, rinsed and mounted with VectaShield (Vector Laboratories Inc., Burlingame, CA). Slides were viewed by fluorescence microscopy and digitally photographed on Nikon Eclipse ME600 microscope with Nikon DXM 1200C camera system at 40X total magnification. The number of Aβ deposits in the cortex and hippocampus was quantified using Nikon NIS Elements Advanced Research Software, version 2.3. The number of Aβ deposits stained with the antibody was measured for each slice using three slices per mouse. Aβ-deposit area fraction (AβAF), which is the total area stained for Aβ counted in a defined region relative to the total area of that region, was calculated by the software.

### Staining for Insulysin and Other Cell Markers

Frozen sections were fixed in acetone for 2 min then blocked for 1 h with 1.5% donkey serum in PBS. Sections were then co-stained for insulysin with goat anti-IDE (1 μg/ml, Sigma) and for the astrocyte marker GFAP with rabbit anti-GFAP (5 μg/ml, DAKO North America Inc., Carpinteria, CA) or for insulysin and a neuronal marker MAP2 (1:1,000, Abcam, Cambridge, MA). Control antibodies from the same companies as the primary antibodies were used in parallel. After rinsing three times in PBS secondary antibodies (Life Technologies) were added for 30 min: Alexa^488^-labeled donkey anti-rabbit (4 μg/ml), Alexa^555^-labeled donkey anti-goat (4 μg/ml). Slides were then rinsed and mounted with VectaMount and digitally photographed via fluorescence microscopy at total magnification of 400X.

### Isolation of CD11b+ Cells

Transgenic PS1-APP, their WT litter mates, PS1-APP/CX3CR1^−/−^ or PS1-APP/CX3CR1^+/−^ were euthanized and perfused with 30 cc PBS without Ca^++^ and Mg^++^ (PBS^=^). Brains were then removed, rinsed in PBS^=^ and placed separately into a C-tube (Miltenyi Biotech, Auburn, CA) with RPMI (no phenol red) containing 2 mM L-glutamine (Mediatech), Dispase (2 U/ml) and 0.2% Collagenase Type 3 (Worthington Biochemicals, Lakewood, NJ) according to our previous protocol (5). Brains were processed using the gentleMACS Dissociator (Miltenyi Biotech) on the brain program settings according to manufacturer's directions. Briefly, the brains were subjected to *three* rounds of dissociation each followed by a period of incubation at 37°C. After the *second* round of dissociation, DNase I grade II (Roche Applied Science, Indianapolis, IN) was added at a concentration of 40 U/ml and incubated for an additional 10 min before the final round of dissociation. The digestion enzymes were inactivated by addition of PBS^=^ containing 2 mM EDTA and 5% fetal bovine serum (FBS) and the digested brain bits were triturated gently and passed over a 100 μm filter (Fisher Scientific, Pittsburgh, PA). Cell pellets were resuspended in 10.5 ml RPMI/L glutamine, mixed gently with 4.5 ml physiologic Percoll® (Sigma Aldrich), and centrifuged at 850 × g for 45 min. The cell pellet was resuspended in PBS^=^ and then passed over a 40 μm filter (Fisher Scientific), centrifuged and washed again with PBS^=^. Microglia were then isolated as previously described (9). The number of cells isolated per hemisphere were recorded as shown in **Figure 2C**. Cells were centrifuged, and the pellets were lysed in RLT-Plus buffer from the RNeasy® Plus mini kit (Qiagen Inc., Valencia, CA) to use for QPCR.

### Co-staining of Brains for CD11b and Aβ Plaques

Frozen sections from PS1-APP, WT littermates, and PS1-APP/CX3CR1^−/−^ or PS1-APP/CX3CR1^+/−^ were fixed in acetone for 2 min, washed in PBS, then treated with 0.25% trypsin for antigen retrieval. Endogenous peroxidase activity was quenched with 0.3% H_2_O_2_ followed by blocking with 1.5% donkey serum in PBS. Sections were incubated overnight at 25°C with rat anti-CD11b (clone 5C6) (AbD Serotec) or rat IgG2b negative control (AbD Serotec) each at 10 μg/ml in PBS with 1.5% donkey serum. The slides were then processed using the Vectastain® Elite ABC reagent (Vector laboratories) according to the manufacturer's instructions followed by development with the NovaRed™ Peroxidase Substrate kit. Aβ-containing plaques were stained with 1% Thioflavin-S (Sigma Aldrich) for 5 min in the dark. Finally, the sections were counterstained with hematoxylin, mounted with VectaMount and digitally photographed via brightfield microscopy to detect CD11b, and via fluorescence to visualize Thioflavin-S. Since different size plaques can exist within any single mouse at any single age, the size of the plaques was determined using Image J and the number of CD11b positive cells associated with plaques ≥75 μm in their largest diameter was quantified by two independent blinded laboratory members. A minimum of 80 plaques were counted per genotype.

### Quantification of Aβ (1-42) and Aβ (1-40)

Brains from transgenic PS1-APP, WT littermates, and PS1-APP-CX3CR1^+/−^ were harvested sans cerebellum, weighed and homogenized in 8X weight/volume of 5M guanidine using the TissueRuptor (Qiagen) homogenizer. Total Aβ (1-42) and Aβ (1-40) were measured in whole brain homogenates using colorimetric immunoassay kit for human Aβ (1-42) and Aβ (1-40) (Life Technologies) according to manufacturer's instructions. Briefly, brain homogenates were diluted and added to wells coated with a monoclonal antibody specific for the NH-terminus of human Aβ and co-incubated with a rabbit antibody specific for the COOH-terminus of Aβ (1-42) or Aβ (1-40)_._ Horseradish peroxidase labeled anti-rabbit IgG is used to detect bound rabbit antibody through use of substrate solution. Amount of Aβ (1-42) and Aβ (1-40**)** in brain samples was determine using a standard curve of known amounts of human Aβ (1-42) or Aβ (1-40) supplied with the kits.

### Quantitative Real Time PCR

Total RNA from CD11b+ cells was isolated using the RNeasy® Plus mini kit for RNA isolation (Qiagen) and RNA from whole brain homogenates was isolated using the RNeasy® Lipid Tissue kit (Qiagen) according to the manufacturer's instructions and quantified using the Nanodrop™ 2000 (Thermo Fisher Scientific, Waltham, MA). RNAs (500 ng−1.5 μg) were reverse transcribed using the RT^2−^first strand kit (SA Biosciences). The qPCR was performed with the MX4000™ unit (Agilent Technologies, Santa Clara, CA) using SYBR Green to detect the amplification products as described (21, 22). The following cycles were performed: initial denaturation cycle 95°C for 10 min, followed by 40 amplification cycles of 95°C for 15 s and 60°C for 1 min and ending with one cycle at 25°C for 15 s. Analysis was performed on the data output from the MX4000™ software (Agilent technologies) using Microsoft Excel XP. Relative quantification of mRNA expression was calculated by the comparative cycle method described by the manufacturer (Agilent technologies). Primer sequences used were as follows: CX3CR1 (forward) ACCGGTACCTTGCCATCGT (reverse) ACACCGTGCTGCACTGTCC. β2 microglobulin (forward) CCGAACATACTGAACTGCTAC (Reverse) CCCGTTCTTCAGCATTTGGA. Insulysin (Forward) GAAGACAAACGGGAATACCGTG (Reverse) CCGCTGAGGACTTGTCTGTG. Neprilysin (Forward) GCAGCCTCAGCCGAAACTAC (Reverse) CACCGTCTCCATGTTGCAGT. MMP9 (Forward) GCCATGCACTGGGCTTAGAT (Reverse) TCTTTATTCAGAGGGAAGCCCTC.

### Behavioral Studies: Barnes Maze

The Barnes maze is a spatial learning task that allows a subject to escape from aversive stimuli, such as bright light, by using environmental cues to locate an escape box. The Barnes maze (Stoelting, Wood Dale, IL) is a light gray circular acrylic table top that is 91 cm in diameter with 20 equally spaced holes that are each 5 cm in diameter and 5 cm from the edge. Under one of the holes an escape box (target box) is mounted that allows an animal to enter and escape while the remaining holes are equipped with false escape boxes that do not allow entry but will remove any cues that may be viewed through open holes. We used a protocol that allowed detection of spatial learning deficits in PS1-APP mice that had been trained first with the cued version followed by the non-cued version (23). Mice show impaired spatial learning in the non-cued, hidden-fixed target version, but not in the cued-variable version. In the cued-variable version of the Barnes maze, the target hole is marked with a conspicuous polystyrene cone next to the target hole on the maze surface and the target location is varied from trial to trial. In the non-cued, hidden target version, the target hole was always located in the same place relative to extra-maze cues, but the maze itself was rotated between trials and thoroughly cleaned to eliminate intra-maze cues such as odor. Using this protocol, we tested WT, PS1-APP, and PS1-APP/CX3CR1 ^−/+^ mice 280–300 days of age. To begin each trial, the subject was placed in the middle of the maze and covered with a black box for 10 s before being released. The mouse was then allowed to explore the maze until it entered the target box up to 180 s. If a mouse did not find the target after 180 s, it was gently guided to the correct hole and allowed to enter. Once in the escape box, the hole was covered, and the mouse was allowed to sit inside the box for 1 min before being gently returned to its home cage. Training sessions comprised three trials per mouse, with a rest interval of 20–25 min between trials. On the fifth day, a single 90-s probe trial was run in which all holes were blocked and the number of pokes into the “target” hole and adjacent holes were counted. The probe trial is performed in order to determine if the mouse remembers the location of the target. Cued-variable target and non-cued, hidden target sessions were run on four consecutive days each. The principle measure was errors time to enter target hole (escape latency). All experiments were videotaped, and the input was analyzed using the AnyMaze software (Stoelting).

### Statistical Analysis

Statistical analysis was performed using student *T*-test or one-way ANOVA with between-groups differences determined by Tukey analysis provided in the “Microcal Origin 8” graphics and statistics software and *p* < 0.05 were considered significant.

## Results

### CX3CR1 Deficiency Is Associated With Reduced Plaque Load and Reduced Aβ Levels in PS1-APP Mice

**Figure 1:**
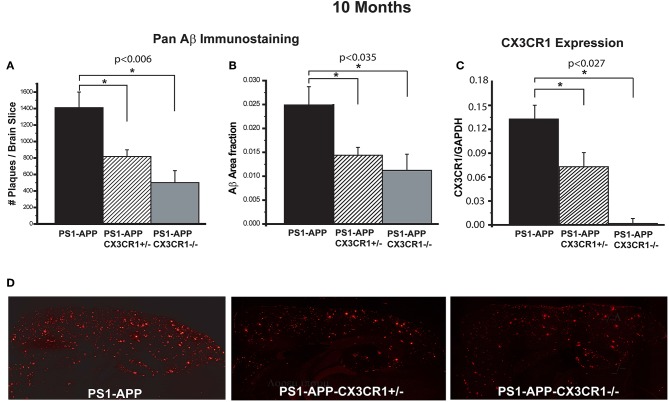
CX3CR1 deficiency results in reduced Aβ deposits and levels of Aβ in 10-month old PS1-APP-CX3CR1-deficient genotypes. **(A–C)** Fixed brain sections from 10-month old PS1-APP, PS1-APP-CX3CR1^+/−^, and PS1-APP-CX3CR1^−/−^ mice were stained using polyclonal pan-Aβ antibody. The number of Aβ deposits **(A)** and Aβ-deposit area fraction **(B)** were determined using three sections per mouse (Values represent group mean ± SEM). **(C)** Quantitative PCR measurements of CX3CR1 RNA in 10-month old PS1-APP, PS1-APP-CX3CR1^+/−^, and PS1-APP-CX3CR1^−/−^ microglia show a gene dosage effect. **(D)** Representative fluorescent micrographs of plaques (red) in PS1-APP, PS1-APP-CX3CR1^+/−^, and PS1-APP-CX3CR1^−/−^ mice. For all measurements in this figure, *n* = 6 for PS1-APP, *n* = 7 for PS1-APP-CX3CR1^+/−^, and *n* = 3 for PS1-APP-CX3CR1^−/−^. * represent statistically significant.

To determine the effect of CX3CR1 deficiency on AD-like pathology in PS1-APP mice, we assessed Aβ load and levels in brains of PS1-APP transgenic mice and PS1-APP-CX3CR1 deficient mice at 10 months of age. In studies to assess Aβ load, fixed-frozen sections were stained with a polyclonal pan-Aβ antibody that recognizes both Aβ (1-42) and Aβ (1-40) in areas of Aβ deposit. The number of Aβ deposits per section (cortex + hippocampus) and Aβ-deposit area fraction (AβAF) were quantified in PS1-APP, PS1-APP-CX3CR1^+/−^, and PS1-APP-CX3CR1^−/−^ mice. Compared with PS1-APP mice, there were significant decreases in the number of Aβ-deposits (Figure 1A) and AβAF (Figure 1B) in both PS1-APP-CX3CR1-deficient genotypes. The number of Aβ deposits per section in PS1-APP-CX3CR1^+/−^ and APP-CX3CR1^−/−^ mice was reduced to 58% (818 ± 81, *p* = 0.006) and 35% (501 ± 144, *p* = 0.007), respectively, of PS1-APP mice (1,409 ± 189). PS1-APP-CX3CR1^−/−^ mice did not have a statistically different number of Aβ deposits than PS1-APP-CX3CR1^+/−^ mice. In addition to reduced Aβ-deposit number, the AβAF was also significantly reduced in PS1-APP-CX3CR1-deficient mice compared to PS1-APP mice. AβAF in PS1-APP-CX3CR1^+/−^ mice was reduced to 60% of PS1-APP values (0.015 ± 0.0017 vs. 0.025 ± 0.0038, *p* = 0.035) and in PS1-APP-CX3CR1^−/−^ mice it was reduced to 44.8% (0.0112 ± 0.0033 vs. 0.025 ± 0.0038, *p* = 0.023). The difference in AβAF of PS1-APP-CX3CR1^+/−^ and PS1-APP-CX3CR1^−/−^ was not statistically significant. Quantitative PCR analysis confirmed that CX3CR1 RNA was not detectable in microglia isolated from PS1-APP-CX3CR1^−/−^ mice and that level of CX3CR1 RNA in microglia from PS1-APP-CX3CR1^+/−^ mice was ~54% of that of microglia from PS1-APP mice confirming a gene dosage effect on CX3CR1 expression (Figure 1C). Figure 1D shows examples of immunofluorescent staining with Pan-Aβ antibody in PS1-APP, PS1-APP-CX3CR1^+/−^, and PS1-APP-CX3CR1^−/−^ brains.

**Figure 2:**
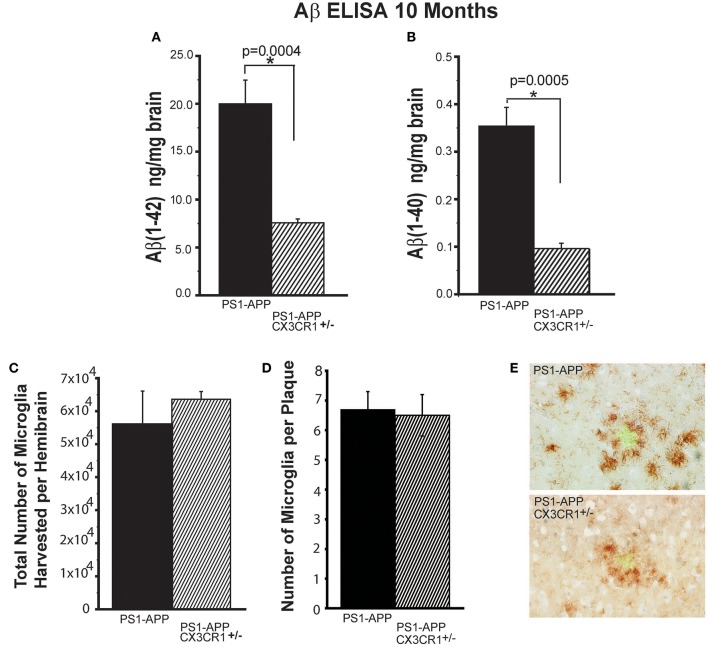
Partial CX3CR1 deficiency affects Aβ levels but does not affect total microglial numbers or the numbers of plaque-associated microglia. Quantification of **(A)** Aβ (1-42) and **(B)** Aβ (1-40) by ELISA in brain homogenates of 10-month old mice (Numbers represent mean ng of Aβ/mg brain ± SEM). **(C)** Quantification of total number of microglia isolated per hemisphere. **(D)** Quantification of the number of CD11b+ cells associated per plaque ≥75 μm in diameter. **(E)** Representative micrographs of plaques (green) and microglia (brown) in PS1-APP and PS1-APP-CX3CR1^+/−^ mice. For all measurements in this figure, *n* = 6 for PS1-APP, *n* = 7 for PS1-APP-CX3CR1^+/−^. For **(E)** 80 plaques were counted per genotype. * represent statistically significant.

Because there were no significant differences in Aβ deposits between PS1-APP-CX3CR1^+/−^ and PS1-APP-CX3CR1^−/−^, we focused subsequent analysis on PS1-APP-CX3CR1^+/−^ mice. The reduction in plaque number and AβAF in PS1-APP-CX3CR1-deficient mice correlated with reduced levels of Aβ (1-42) and Aβ (1-40) in brain homogenates as determined by a commercial ELISA kit (Figures 2A,B). There was a 2.6-fold decrease in Aβ (1-42) in PS1-APP-CX3CR1^+/−^ mice compared with PS1-APP mice (Figure 2A) (7.56 ng/mg brain ± 0.41 vs. 19.8 ± 2.6 ng/mg brain, *p* = 0.0004. Levels of Aβ (1-40) were also significantly reduced in PS1-APP-CX3CR1^+/−^ mice (Figure 2B). Aβ (1-40) levels were 3.5-fold lower in PS1-APP-CX3CR^+/−^ brain homogenates compared with PS1-APP brains (0.10 ± 0.011 vs. 0.35 ± 0.039 ng/mg brain, *p* = 0.0005). These data indicate that partial CX3CR1 deficiency in heterozygous PS1-APP-CX3CR^+/−^ is associated with a significant reduction in Aβ deposits and load and in the levels of Aβ (1-42) and (1-40) compared to PS1-APP mice and that complete deletion of CX3CR1 is not necessary to confer beneficial effects on AD-like pathology in these mice.

### Partial CX3CR1 Deficiency Does Not Affect Microglial Number and Association With Plaques of the Same Size

To determine if partial CX3CR1 deficiency affects overall microglial numbers in the brain, we quantified the total number of microglia harvested per hemisphere of PS1-APP-CX3CR1^+/−^ and PS1-APP mice and found no statistically significant differences between the two genotypes (Figure 2C). We also compared the numbers of CD11b+ cells associated per plaque and found that when comparing same size plaques (≥75 μm) there were no statistically significant differences between the two genotypes (Figures 2D,E). These data show that partial CX3CR1 deficiency does not affect the total number of microglia or accumulation of microglia at sites of Aβ accumulation.

### Partial CX3CR1 Deficiency in PS1-APP-CX3CR1^+/−^ Mice Is Associated With Reduced Aβ Load as Early as 5 Months of Age and Continues to 24 Months

**Figure 3:**
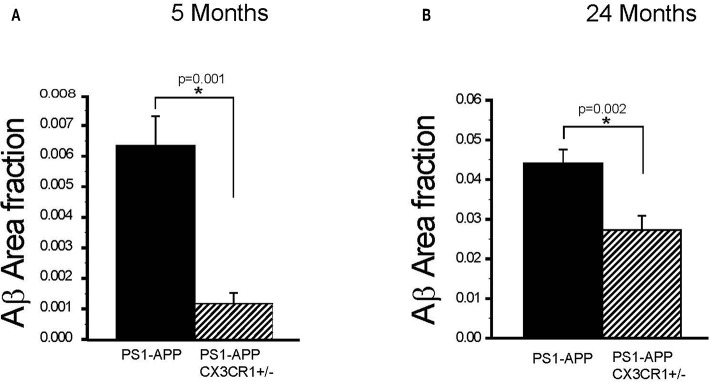
CX3CR1 deficiency results in reduced plaque-load in 5- and 24-month old PS1-APP-CX3CR1^+/−^ mice. **(A,B)** Brain sections from PS1-APP and PS1-APP-CX3CR1^+/−^ mice at 5 and 24-months of age were stained and Aβ area fraction (AβAF) was determined for hippocampus + cortex in mice at 5 and 24-months of age. **(A)** At 5 months of age AβAF is significantly reduced in PS1-APP-CX3CR1^+/−^ (*n* = 3) mice compared with PS1-APP mice (*n* = 8). **(B)** At 24- months of age, AβAF is also significantly reduced in PS1-APP-CX3CR1^+/−^ (*n* = 5) mice compared with PS1-APP mice (*n* = 6). For all groups three sections per mouse were quantified and numbers represent group mean ± SEM. * represent statistically significant.

To determine if the effect of partial CX3CR1 deficiency on Aβ deposition and levels starts early and persists at advanced stages of disease in PS1-APP mice, we assessed the effect of CX3CR1 deficiency on AβAF in brains of PS1-APP and PS1-APP/CX3CR1^+/−^ mice at 5 and 24 months of age. In 5-month old animals there was a significant 5.5-fold reduction in AβAF (Figure 3A) in PS1-APP-CX3CR1^+/−^ compared with PS1-APP mice (0.0011 ± 0.0003 vs. 0.0061 ± 0.0009, respectively, *p* = 0.001).

Similarly, at 24-months of age there were significant decreases in AβAF in -APP-CX3CR1^+/−^ (38% reduction, 0.027 ± 0.003, *p* = 0.02) compared with PS1-APP brains (0.044 ± 0.0034) (Figure 3B). These data indicate that partial CX3CR1 deficiency slows the accumulation of Aβ in PS1-APP mice starting early in the disease process and continues to do so at late disease stages.

### CX3CR1 Deficiency Is Associated With Increased IDE Levels in PS1-APP-CX3CR1^+/−^ Mice

**Figure 4:**
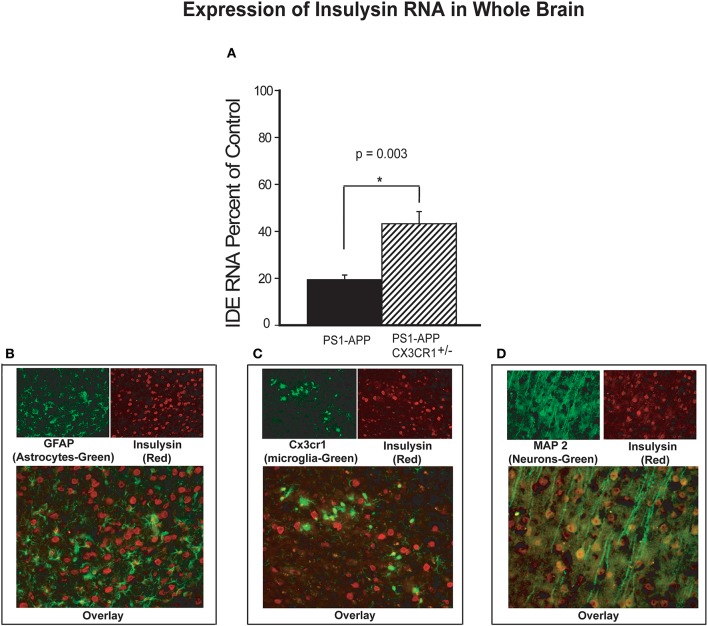
Increased levels of insulysin RNA in brains of CX3CR1-deficient PS1-APP transgenic mice. **(A)** RNA was extracted from brains of PS1-APP, WT, PS1-APP-CX3CR1^+/−^, and CX3CR1^+/−^ mice and assessed for expression of insulysin (IDE) by qPCR. Data represent mean percent of appropriate control ± SEM (*n* = 6 per genotype). **(B–D)** CX3CR1-dficient or WT mouse brain sections were co-stained for IDE and cell-specific markers and photographed by fluorescence microscopy at 40X magnification. **(B)** WT brain section co-stained for the astrocyte marker GFAP (green cells), for IDE (red cells). Most of the cells stained for IDE do not express GFAP. **(C)** CX3CR1-deficient brain section showing GFP-expressing microglia (green) and stained for IDE (red cells). Most of the microglia do not show detectable levels of IDE. **(D)** WT mouse brain slices co-stained for a neuronal marker MAP2 (green cells) and IDE (red cells). Most of the MAP2-expressing cells (neurons) also express IDE. * represent statistically significant.

Aβ levels in the brain are regulated in part by several Aβ-degrading enzymes, including insulin degrading enzyme (IDE-insulysin), neprilysin and matrix metalloprotease 9 (MMP9) (4). Progression of Alzheimer's disease in humans and mouse models of the disease is associated with reduced expression of these Aβ-clearing enzymes resulting in decreased Aβ clearance and increased Aβ accumulation (5, 24). Because partial CX3CR1 deficiency is associated with lower Aβ levels and decreased plaque load in PS1-APP mice, we hypothesized that under pathological conditions such as AD, CX3CR1 may regulate expression of Aβ degrading enzymes. To test this hypothesis, we measured RNA levels of IDE, neprilysin, and MMP9 in brain homogenates of PS1-APP, their wild type (WT) littermate controls, PS1APP-CX3CR1^+/−^ mice and their CX3CR1^+/−^ littermate controls. IDE RNA (expressed as percent of appropriate littermate controls), was reduced in brains of PS1APP to 19.0% (±2.0) of control level while in PS1-APP-CX3CR1^+/−^ it was reduced to only 43% (±5.1) of control (Figure 4A). The differences in expression of IDE RNA between PS1-APP mice and PS1-APP-CX3CR1^+/−^ mice was significant (*p* = 0.003). These data indicate that there is an inverse relationship between CX3CR1 and IDE expression and suggest that partial CX3CR1 deficiency upregulates expression of IDE.

To identify the brain cells that predominantly expressed IDE, fixed frozen sections from CX3CR1deficient brains were stained for IDE and sections from WT mice were co-stained for IDE and MAP2 (neuronal marker) or for IDE and GFAP (astrocyte marker). Co-staining for IDE (red) and GFAP (green, Figure 4B) showed very little co-staining of IDE in GFAP containing cells and intense staining for IDE in cells lacking GFAP. In Figure 4C, GFP-positive microglia are seen evenly distributed or as small clusters, but IDE was much less detectable in microglia compared with intense positive staining seen in other cells throughout the brain. In contrast, co-staining for IDE (red) and MAP2 (green) shows intense positive stain for IDE in neurons containing MAP2 (yellow, Figure 4D). It is possible that detection of IDE protein in microglia and astrocytes is limited by the sensitivity of the staining assay. However, our data strongly suggests that the bulk of IDE expression in the brain occurs in neurons.

### Effect of CX3CR1 Deficiency on Levels of Neprilysin and MMP9 in Brains of PS1-APP/CX3CR1-Deficient Mice

In addition to IDE, Aβ levels in the brain can be regulated by neprilysin, another important Aβ-degrading enzyme. To determine if CX3CR1 deficiency is also associated with up-regulation of neprilysin levels similar to what we observed with IDE, we measured expression of neprilysin in brains of PS1-APP, their WT littermate controls, PS1APP-CX3CR1^+/−^ mice, and their controls in the brain. In contrast to what we observed with IDE, we did not find any significant differences in neprilysin expression in the brains of PS1-APP and PS1-APP/CX3CR1^+/−^ mice (Data not shown).

**Figure 5:**
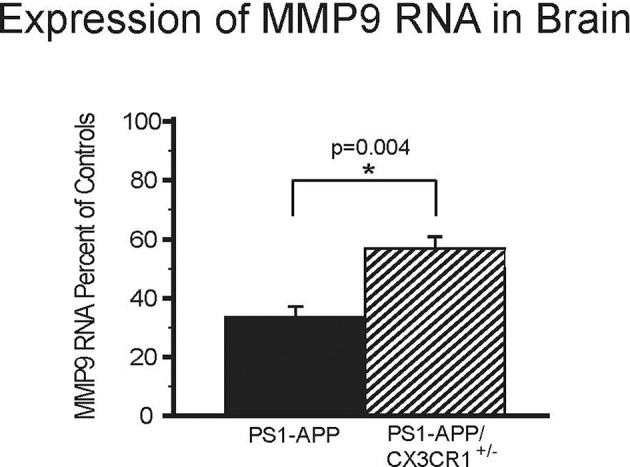
Effects of CX3CR1-deficiency on levels of MMP9 RNA in the whole brains of PS1-APP mice. RNA was extracted from brains of PS1-APP, WT, PS1-APP-CX3CR1^+/−^, and CX3CR1^+/−^ mice and assessed for expression of MMP9 by qPCR. Data are expressed as mean percent of appropriate control ± SEM (*n* = 6 per genotype). * represent statistically significant.

MMP9 is an important Aβ-degrading enzyme expressed on microglia (25) and neurons (26). To determine if CX3CR1 deficiency is also associated with up-regulation of MMP9 levels similar to what we observed with IDE, we measured MMP9 RNA in the brains of PS1-APP, PS1APP-CX3CR1^+/−^, and their CX3CR1^+/−^ littermates. There were statistically significant differences in MMP9 expression in PS1-APP-CX3CR1^+/−^ compared with PS1-APP mice (Figure 5). PS1-APP mice MMP9 levels fell to 33.6% (±3.5) of controls, while PS1-APP/CX3CR1^+/−^ mice MMP9 expression was only reduced to 56.9% (±3.9) of controls. Compared with PS1-APP mice, MMP9 levels in PS1-APP/CX3CR1^+/−^ were 69% higher (*p* = 0.004). These data indicate that, similar to IDE, partial CX3CR1-deficiency mitigates the reduction in MMP9 observed in aging PS1-APP mice.

### CX3CR1 Deficiency Is Associated With Increased Levels of Aβ-Degrading Enzymes in Brain Homogenates of PS1-APP-CX3CR1^+/−^ Mice but Not in Their Microglia

**Figure 6:**
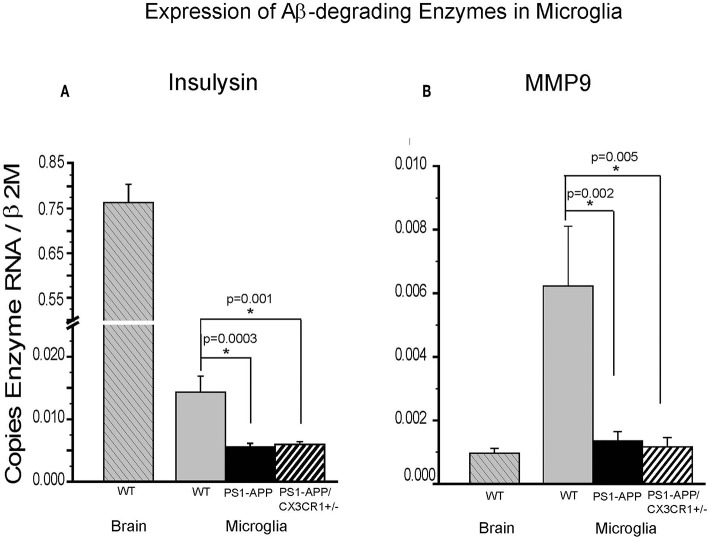
Effects of CX3CR1 deficiency on expression of IDE and MMP9 in microglia from WT, PS1-APP, and PS1-APP-CX3CR1^+/−^ mice. Expression of IDE **(A)** MMP9 **(B)** was analyzed by qPCR on RNA isolated from microglia freshly harvested from brains of PS1-APP, WT littermates, and PS1-APP-CX3CR1^+/−^ mice at 24 months of age: WT brain (*n* = 6), WT microglia (*n* = 6), PS1-APP (*n* = 10), and PS1-APP-CX3CR1^+/−^ (*n* = 6). Levels of enzymes are expressed relative to the β2M housekeeping gene and bars represent mean ± SEM. * represent statistically significant.

We have previously shown that expression of IDE in microglia from PS1-APP mice decreases with age and progression of AD-like pathology (5). To determine if the significant preservation of IDE in PS1-APP-CX3CR1-deficient mice occurs in microglia as well as in whole brain, we measured IDE (Figure 6A) and MMP9 (Figure 6B) expression in freshly isolated microglia from 24-month old WT, PS1-APP, and PS1-APP-CX3CR1^+/−^ mice. IDE RNA levels in PS1-APP microglia were significantly reduced to 42% of WT control microglia (0.0056 ± 0.00055 vs. 0.014 ± 0.002, *p* = 0.0003). IDE expression in PS1-APP/CX3CR1^+/−^ microglia was also significantly decreased to 45% of WT control levels (0.0063 ± 0.00039 vs. 0.014 ± 0.002, *p* = 0.001). In contrast to findings in whole brain where IDE levels in PS1-APP/CX3CR1^+/−^ mice were significantly higher than in PS1-APP mice, IDE expression in microglia from PS1-APP/CX3CR1^+/−^ remained at the lower levels seen in PS1-APP mice. These data indicate that CX3CR1 deficiency in microglia results in preservation or maintenance of IDE levels in brain cells other than microglia. IDE expression in microglia from WT mice was 54-fold lower than in whole brains from mice of the same age, suggesting that the bulk of IDE expressed in the brain of 2-year old mice occurs in cells other than microglia.

In contrast, expression of MMP9 in WT microglia was 6-fold higher than in whole brain (0.0062 ± 0.0018 vs. 0.00097 ± 0.00015, respectively) indicating that MMP9 is highly expressed in microglia but is also expressed in other cells of the brain. Expression of MMP9 in microglia from PS1-APP mice and PS1-APP/CX3CR1^+/−^ mice was significantly decreased to 23% and 20% of WT microglia (0.0014 ± 0.00029 and 0.0012 ± 0.00029 vs. 0.006 ± 0.0019), respectively. These data indicate that microglial CX3CR1 deficiency did not preserve expression of Aβ degrading enzymes in microglia, and that the preservation of MMP9 observed in mouse brains was due to the effects of CX3CR1-deficiency on cells other than microglia.

### CX3CR1 Deficiency Is Associated With Improved Barnes Maze Function in PS1-APP-CX3CR1^+/−^ Mice

**Figure 7:**
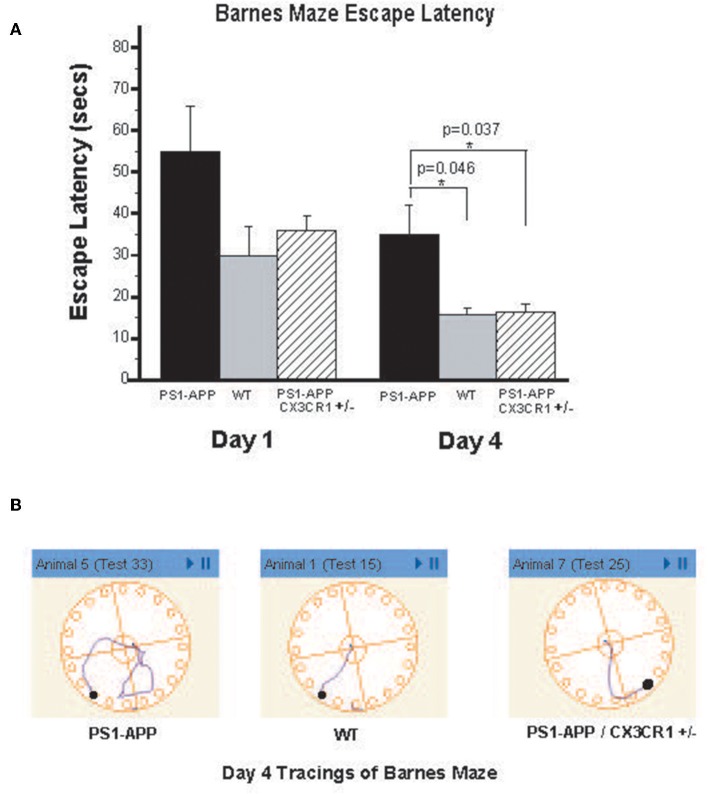
PS1-APP-CX3CR1^+/−^ mice perform better that PS1-APP in the Barnes maze behavioral test. Mice at 10-months of age were first trained for 5 days on cued-target Barnes maze and then data collected during a 4-day training using hidden-fixed target protocol on Barnes maze. Escape latency, which is the time for a subject to locate and enter the escape box, was collected for each mouse during all trials and is shown in **(A)** for Days 1 and 4 of the hidden-fixed target Barnes maze protocol (mean escape latency in s ± SEM). On Day 1 there were no significant differences in escape latencies between genotypes. By Day 4, PS1-APP animals took significantly longer to escape the maze compared with both WT and PS1-APP-CX3CR1^+/−^ mice. **(B)** Shows representative maze tracings for each genotype on Day 4 of the hidden-fixed target protocol. Animal numbers are WT (*n* = 6), PS1-APP (*n* = 8) and PS1-APP-CX3CR1^+/−^ (*n* = 6). * represent statistically significant.

To determine if the observed reduction in brain Aβ levels is associated with improved cognitive and memory functions in PS1-APP-CX3CR1^+/−^ deficient mice, we tested visuo-spatial learning and memory in these mice using the Barnes maze. PS1-APP, PS1-APP/CX3CR1^+/−^, and WT littermate mice were tested at 300 days of age as detailed in materials and methods. Figure 7A shows results of escape latency (time to find target and hide in it) for the non-cued hidden fixed arm of the study on the four training days. On Day 1 there were no significant differences in the escape latencies between PS1-APP, PS1-APP-CX3CR1^+/−^, and WT mice. However, by Day 4, while all mice groups showed improvement in the escape latency, there were statistically significance decreases in both WT (15.57 ± 1.66, *p* < 0.05) and PS1-APP-CX3CR1^+/−^ mice (16.27 ± 2.06, *p* < 0.05) escape latency compared to PS1-APP mice (34.9 ± 7.13 s). Figure 7B shows representative Day 4 tracings from mice in all three groups. WT and PS1-APP-CX3CR1^+/−^ mice navigated to the escape box quickly, while the PS1-APP mice tended to meander and hesitated longer before identifying the correct hole with the escape box. A similar trend was observed when the mice were tested in the T maze, another test for spatial learning, but the differences did not reach statistical significance (data not shown). To determine if functions other than visuo-spatial learning and memory were different between the three groups, we used the open field test which measures exploratory behavior independent of memory or learning. As expected (27), PS1-APP, PS1-APP-CX3CR1^+/−^, and WT littermate mice showed no difference in exploratory behavior (data not shown).

## Discussion

This manuscript highlights three important findings. First, we show that partial CX3CR1 deficiency in PS1-APP-CX3CR1^+/−^ mice is associated with significant reductions in the amount of Aβ (1-42) and (1-40) and the number of visible Aβ deposits in their brains compared to regular PS1-APP mice. Complete deletion of CX3CR1 in models of amyloid deposition have been shown to reduce Aβ deposits and enhanced microglial Aβ phagocytosis (16, 17). However, such complete deletion has also been associated with worsening memory deficits and tau pathology (28–31). Our findings show that partial CX3CR1 deletion restores the beneficial effects of lowering Aβ levels and show improvement in behavioral/cognitive testing. It remains to be seen whether partial CX3CR1 deletion affects tau pathology. However, in combined models of Aβ and tau pathology, Aβ deposition precedes and seems to drive tau pathology. We are currently exploring the effects of partial CX3CR1 deletion on disease progression in one of these combined models. Importantly, it is unlikely that pharmacologic targeting of CX3CR1 will achieve the effects of complete deletion of this receptor as in CX3CR1^−/−^ mice. However, it is possible to achieve partial inhibition of CX3CR1 activity pharmacologically, similar to what is observed in CX3CR1^+/−^ mice heterozygous for this receptor suggesting that the beneficial effects of partial blockade of CX3CR1 function are preserved while mitigating the harmful effects of such blockade.

The second important finding is that partial CX3CR1 deficiency is associated with altered gene expression of neuronal Aβ degrading enzymes. Microglia do not exist statically in the brain but actively patrol their immediate environment by constant extension and retraction of dendritic-like arms (31). During the course of their normal functioning they make contact with the surfaces of other cells, including neurons. Given the paired expression of CX3CL1 (fractalkine) on neurons and CX3CR1 on microglia in the brain, it has been proposed that the interactions between neurons and microglia mediated through the fractalkine-CX3CR1 complex may modulate microglia functions (30). Our data suggest that a reciprocal interaction may also be occurring and that microglia may regulate neuronal function and gene expression profile via the fractalkine-CX3CR1 pathway. Indeed, by examining the gene expression profile of whole brains and of purified microglia, we found that partial microglial CX3CR1 deficiency reverses or slows the reduction in insulysin and MMP9 Aβ-degrading enzymes observed in PS1-APP mice as they age, and their AD-like pathology progresses. For insulysin, such restoration appears to occur exclusively in neurons, since IDE is expressed predominantly in neurons as evidenced by immuofluorescence staining of brain slices. Similarly, when we evaluated the Aβ-degrading enzyme MMP9, which is expressed in microglia and neurons, we also found reduced expression with aging and AD progression, CX3CR1 deficiency did not restore expression of this enzyme to WT levels in microglia but there was restoration of MMP9 in whole brain. These observations are not explained by a decrease in microglial number since the total number of microglia in the brains of PS1-APP and PS1-APP-CX3CR1^+/−^ were similar (not shown). These findings indicate that microglial CX3CR1 regulates expression of neuronal Aβ degrading enzymes. Because the only known ligand for microglial CX3CR1 is neuronal fractalkine, these findings suggest that interactions of microglial CX3CR1 with neuronal fractalkine regulate gene expression in neurons.

Heterozygous CX3CR1^+/−^ mice have been used in lieu of WT mice when studying the effects of complete deletion of CX3CR1 or when using CX3CR1-CRE mice. The third important finding is that our data show that, at least in the case of models of Aβ deposition, CX3CR1^+/−^ are clearly different that CX3CR1^+/+^ mice and that partial CX3CR1 deficiency significantly alters microglial functions.

It is important to note when interpreting our findings is that due to limited availability of mice of different genotypes, these experiments were performed in male mice. It is to be determined if female mice will exhibit similar differences between the genotypes.

Collectively, our data indicate that partial CX3CR1 deficiency slows progression of AD-like pathology in a transgenic mouse model of the disease and reverses cognitive deficit, by restoring neuronal Aβ-degrading pathways possibly by disrupting CX3CR1-fractalkine interactions. Partial down-regulation of microglial CX3CR1 expression, and/or inhibition CX3CR1-fractalkine interactions should be explored as a potential therapeutic target to delay disease progression and improve cognition in AD.

## Data Availability Statement

The datasets generated for this study are available on request to the corresponding author.

## Ethics Statement

All protocols were approved by the Massachusetts General Hospital Institutional Animal Care and Use Committee and met US National Institutes of Health guidelines for the humane care of animals.

## Author Contributions

SH designed the study, performed and co-analyzed the experiments, and co-wrote the manuscript. EA, UC, and NK-G performed experiments and co-analyzed data. JE conceived the study, designed the experiments, co-analyzed the data, and co-wrote the manuscript.

### Conflict of Interest

The authors declare that the research was conducted in the absence of any commercial or financial relationships that could be construed as a potential conflict of interest.
